# On the isomeric purity of endcap molecules in cholesteric liquid crystal oligomers for near-infrared thermochromic coatings

**DOI:** 10.1080/02678292.2024.2350046

**Published:** 2024-05-26

**Authors:** Henk Sentjens, Janneke M.A. Bloemers, Johan Lub, Carmen Luengo Gonzalez, Augustinus J.J. Kragt, Albert P.H.J. Schenning

**Affiliations:** a Stimuli-Responsive Functional Materials and Devices (SFD), Department of Chemical Engineering and Chemistry, Eindhoven University of Technology (TU/e), Eindhoven, The Netherlands; b Institute for Complex Molecular Systems (ICMS), Eindhoven University of Technology (TU/e), Eindhoven, The Netherlands; c ClimAd Technology, Nijmegen, The Netherlands

**Keywords:** Cholesteric liquid crystals, liquid crystal oligomer, thermochromic materials, isomeric effects, smart windows, near-infrared reflector

## Abstract

Structurally coloured responsive materials provide an interesting avenue for the development of autonomous temperature regulating window films. One interesting class of such thermochromic materials is cholesteric liquid crystals. However, cholesteric liquid crystals have rarely been applied in coatings for smart window applications. In this work, we report the synthesis of endcapped cholesteric liquid crystal oligomers and its application as near-infrared thermochromic coatings for windows. Two isomerically pure monoacrylate endcapping molecules and its isomeric mixture are synthesised. The molecules are used to synthesise a variety of endcapped cholesteric liquid crystal oligomers to study the effect of the isomeric purity on the thermochromic properties of the coatings. It is found that while the oligomers are almost identical in composition and phase behaviour, only one isomer produces a clear transparent coating, highlighting the significance of minute isomeric differences. Remarkably, the thermochromic behaviour of the coatings for all oligomers is the same. The best performing oligomer is able to reversibly blueshift by 250 nanometres when heated from room temperature to 100°C, opening the way of cholesteric liquid crystals for use in temperature regulating window films.

## GRAPHICAL ABSTRACT

**Figure:**
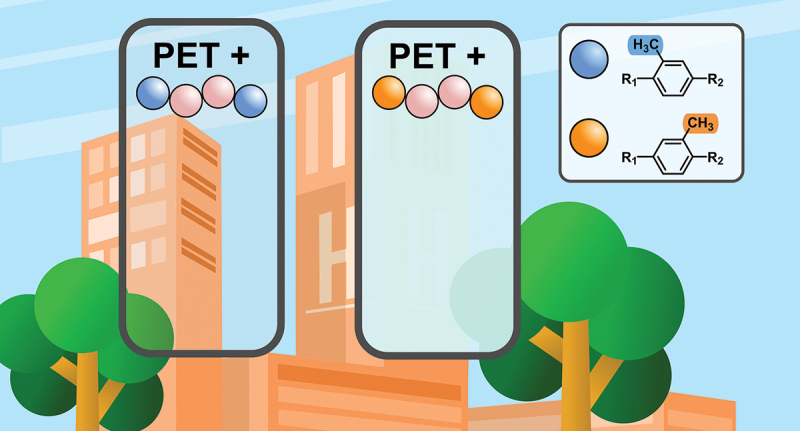

## Introduction

Recently, there has been increasing interest in temperature responsive materials exhibiting structural colours. Structurally coloured materials possess a colour not because of a pigment, but because they form a layered nanostructure that inherently reflects specific wavelengths of light [1]. This is seen in nature within the vibrant foliage of a peacock or the brightly coloured wings of the morpho butterfly, amongst other examples [2,3]. As this colour is tied to the material structure itself, they are stable in time and do not bleach. This makes structurally coloured materials attractive for a wide variety of applications [4–7], including temperature-regulating window films [8–11]. As it is estimated almost 40% of all energy consumption is used for thermal control in buildings [12], and the energy consumption for cooling is expected to triple by 2050 as a result of increasing global temperatures [13], such films are promising to save energy. Temperature-regulating films are designed to autonomously prevent solar near-infrared (NIR) light from entering buildings. This can save from 10% to 30% of the energy spent on indoor temperature management to maintain comfortable living and working spaces [10,14,15]. The films can significantly benefit from an adaptive response to the environmental temperature, allowing solar infrared radiation to pass on cool days while preventing it from entering through a window on hot days [16–22]. Temperature responsive reflective systems based on structural colour are attractive to selectively reflect infrared radiation without affecting the visible light transparency of the window [10,23–25].

One material that can be used to produce dynamic structural colour is liquid crystals (LCs) [26–30]. LCs have a phase in between the solid and liquid state that possess both long-range order seen in crystalline solids and free-flowing properties observed in liquids. The often rod-like molecules can align themselves into a variety of different structures. For example, they can align into the smectic phase, where there exists directional order in addition to a defined layer structure, or into the nematic phase, where the directional order is maintained, but the positional order has disappeared. When a chiral dopant is added to a liquid crystalline material in the nematic phase, the molecules realign into a structurally coloured chiral nematic or cholesteric phase. In this latter phase, the molecular layers are stacked in a helical fashion. This helix functions as a reflector for selective wavelengths, proportional to the distance needed for one full helical rotation, known as the pitch. The helical pitch is ordinarily determined by the concentration of the added chiral dopant in a linear fashion [31]. By making use of the rich phase behaviour exhibited by LCs, it is possible to make the structural colour temperature dependent. For example, when a liquid crystalline material is heated from its smectic to its cholesteric phase, the material changes from a non-reflective to a reflective state. If a material with a gradual rather than a sharp transition from the smectic to the cholesteric phase is used, the cholesteric helix forms gradually across the broad transition, winding up with increasing temperature and shortening the reflected wavelength until the LC becomes fully cholesteric [32]. Such a gradual hypsochromic shift can be achieved by using cholesteric LC oligomer [33].

By making use of diacrylate LCs and dithiol spacer molecules, liquid crystal oligomers can be easily synthesised in a one-pot reaction. Previously, such oligomers were used to produce coatings that selectively reflect infrared light [34,35]. It was demonstrated that monoacrylate LC could be used to control the length and polydispersity of the oligomers (Figure S1) [36]. However, it was found that common LC monoacrylates resulted in structural coloured coatings with poor stability, while new monoacrylate LCs synthesised in nine steps resulted in thermochromic coatings that remained stable over time.

In this work, we synthesised three novel endcapping molecules in four steps while maintaining the desired thermochromic properties exhibited by the oligomers synthesised using these molecules. One synthesised monoacrylate mesogen is an isomeric mixture, while the other two mesogens are its two constituent isomers individually. Remarkably, when the endcapping molecules are used to synthesise oligomers for thin-film coatings, it is found that the isomeric purity of the endcapping molecule plays a significant role in the alignment behaviour of the material: one isomer results in a well-defined cholesteric reflector, while the other becomes scattering and focal conic.

## Experimental

### Materials

2-methyl-1,4-phenylene bis(4-((6-(acryloyloxy)hexyl)oxy)benzoate) (**4**) was purchased from Daken Chemical. ((3 R,3aR,6S,6aR)-hexahydrofuro[3,2-b]furan-3,6-diylbis(4-((4-(((4-(acryloyloxy)butoxy)carbonyl)oxy)benzoyl)oxy)benzoate) (**5**) was purchased from BASF. A 1:1 isomeric mixture of 2-methyl-4-((tetrahydro-2 H-pyran-2-yl)oxy)phenol and 3-methyl-4-((tetrahydro-2 H-pyran-2-yl)oxy)phenol (**6**) was prepared according to a previous publication [37]. 4-hydroxy-3-methylphenyl 4-(3-(acryloyloxy)propoxy)benzoate (**11**) was prepared according to our previous publication [36]. 4-hydroxy-3-methylphenyl 4-(hexyloxy)benzoate (**12**) was prepared according to a previous publication [38]. Surface levelling agent Byk-361N was purchased from BYK-Chemie. All solvents were purchased from BioSolve. All other chemicals were purchased from Sigma-Aldrich. Stretched polyethylene terephthalate (PET) foils with a thickness of 50 μm were purchased from Toyobo Film Solutions.

### Characterization

^1^H-Nuclear Magnetic Resonance (^1^H-NMR) samples are prepared from each oligomer mixture by pipetting 100 μl of the reaction mixture into a separate vial, evaporating the solvent at 50°C for 24 h, and dissolving the dry material into 500 μl of deuterated chloroform. Spectra of the dry products and precursors of the monoacrylate synthesis are produced by dissolving 3 mg of the dry material into 500 μl of deuterated chloroform. Spectra are measured using a Bruker Avance Core iii 400 MHz spectrometer in deuterated chloroform with tetramethyl silane (TMS) used as internal standard. The data are analysed using MestRenova.

Gel Permeation Chromatography (GPC) data is collected using a Shimadzu Prominence-i LC2030C 3D Liquid Chromatograph. GPC samples are prepared from each oligomer by pipetting 30 μl of the respective reaction mixtures into separate vials and evaporating DCM at 50°C for 24 h. The material is weighed, after which THF is added to create a stock solution of 1 mg/ml. One milliliter of this solution is passed through a 2 μm filter and the measurement is performed.

Differential Scanning Calorimetry (DSC) measurements are performed with a TA instruments Q2000. DSC is measured by exposing approximately 5 mg of material to a 5°C/min temperature gradient. The samples are cycled between −50°C and 150°C three times, after which the final measurement is used to determine the transition temperatures. The transition temperatures are rounded to the nearest integer.

Matrix-assisted laser desorption/ionisation time-of-flight mass spectrometry (MALDI-TOF MS) was performed on a Bruker Autoflex Speed MALDI-MS instrument using CHCA (α-cyano-4-hydroxycinnamic acid) as a matrix.

## Synthesis of monoacrylate mesogens

**Scheme 1.**
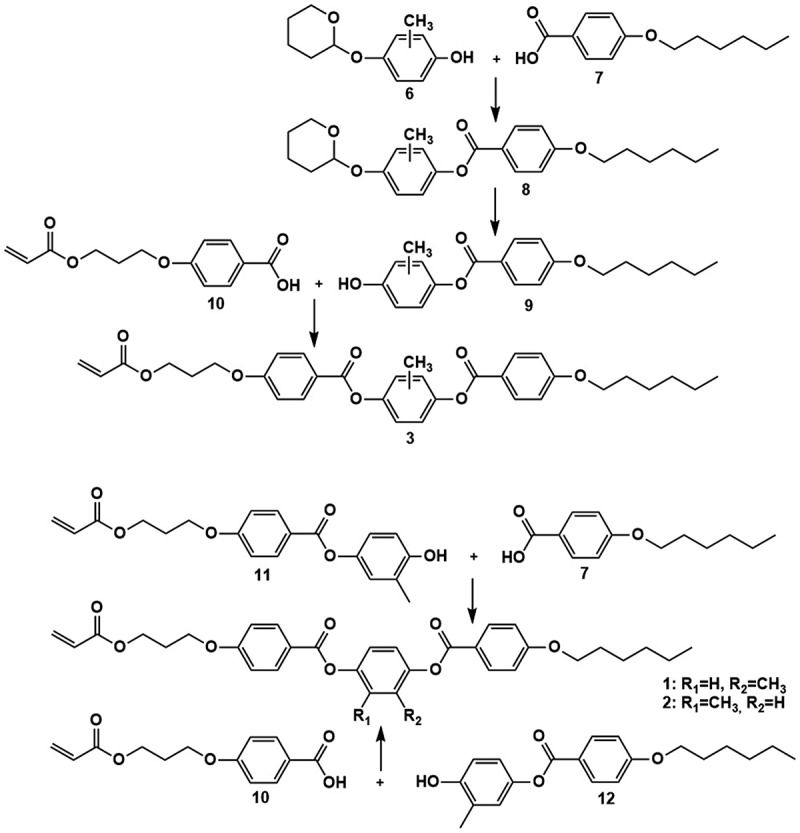
Schematic procedure for the preparation of the monoacrylate compounds **1**, **2**, and **3**.

The schematic representation of the synthesis route for all three isomeric monoacrylate compounds is shown in Scheme 1.

Synthesis of an isomeric mixture of 4-hydroxy-2-methylphenyl 4-(hexyloxy)benzoate and 4-hydroxy-3-methylphenyl 4-(hexyloxy)benzoate (**9**).

45.4 g of N,N’-dicyclohexyl carbodiimide (0.22 mol) was added in portions to a solution of an isomeric mixture of 46 g of 2-methyl-4-((tetrahydro-2 H-pyran-2-yl)oxy)phenol and 3-methyl-4-((tetrahydro-2 H-pyran-2-yl)oxy)phenol (**6**, 0.22 mol), 48.9 g of 4-hexyloxybenzoic acid (**7**, 0.22 mol) and 2.7 g of 4-N,N-dimethylaminopyridine (22 mmol) in 450 ml of dichloromethane, stirred in an ice bath under a nitrogen atmosphere. After stirring for another 16 h at room temperature, the mixture was filtered through a thin silica pad and evaporated. The intermediate isomeric mixture of 2-methyl-4-((tetrahydro-2 H-pyran-2-yl)oxy)phenyl 4-(hexyloxy)benzoate and 3-methyl-4-((tetrahydro-2 H-pyran-2-yl)oxy)phenyl 4-(hexyloxy)benzoate (**8**) obtained as a yellow oil was stirred for 2 h in 400 ml of ethanol at 55°C to which 5.0 g (20 mmol) of pyridinium-4-toluene sulphonate was added. After the addition of 500 ml of water and 250 ml of dichloromethane, the dichloromethane layer was separated, extracted twice with 250 ml of water, once with 200 ml of brine, dried over magnesium sulphate, passed through a thin silica pad, and evaporated. Fifty-four grams (75% yield) of isomeric mixture **9** was obtained as a sticky light brown material, slightly contaminated, but pure enough for the next reaction step. ^1^H-NMR (CDCl_3_, 400 MHz, δ in ppm, J in Hz): 8.15 + 8.12 (d, J = 8.9, 2 H), 6.97 + 6.95 (d, J = 8.9, 2 H), 6.88* + 6.62^#^ (d, J = 8.6, 1 H), 6.90^#^ +6.60* (d, J = 2.7, 1 H), 6.80^#^ +6.56* (dd, J_1_ = 2.8, J_2_ = 8.6, 1 H), 6.2 (br. 1 H), 4.03 (t, J = 6.5, 2 H), 2.18^#^ +2.11* (s, 3 H). 1.81 (p, J = 6.7, 2 H), 1.47 (m, 2 H), 1.3–1.4 (m, 4 H) and 0.92 (t, J = 7.0, 3 H). ^#^signals belong to 4-hydroxy-3-methylphenyl 4-(hexyloxy)benzoate and *signals belong to 4-hydroxy-2-methylphenyl 4-(hexyloxy)benzoate.

Synthesis of the isomeric mixture of 4-((4-(3-(acryloyloxy)propoxy)benzoyl)oxy)-2-methylphenyl 4-(hexyloxy)benzoate and 4-((4-(3-(acryloyloxy)propoxy)benzoyl)oxy)-3-methylphenyl 4-(hexyloxy)benzoate (**3**).

About 33.9 g (0.16 mol) of N,N’-dicyclohexyl carbodiimide was added in portions to a solution of 41.1 g of 4-(3-acryloyloxypropyloxy) benzoic acid (**10**, 0.16 mol), 54.0 g of a mixture of 4-hydroxy-2-methylphenyl 4-(hexyloxy)benzoate and 4-hydroxy-3-methylphenyl 4-(hexyloxy)benzoate (**9**, 0.16 mol) and 1.95 g (16 mmol) of 4-N,N-dimethylaminopyridine in 400 ml of dichloromethane, stirred in an ice bath under a nitrogen atmosphere. After stirring for another 16 h at room temperature, the mixture was filtered through a silica pad and evaporated. About 63.1 g (yield 70%) of isomeric mixture **3** was obtained as a white powder after crystallisation from ethanol and drying over silica in a vacuum desiccator. mp = 67°C and cp = 153°C.

^1^H-NMR (CDCl_3_, 400 MHz, δ in ppm, J in Hz): 8.17 + 8.16 + 8.15 + 8.14 (d, J = 8.8, 4 H), 7.18 (d, J = 8.6, 1 H), 7.13 (d, J = 2.7, 1 H), 7.08 (dd, J_1_ = 8.6, J_2_ = 2.7, 1 H), 7.00 + 6.99 + 6.98 + 6.97 (d, J = 8.8, 4 H), 6.43 (dd, J_1_ = 17.3, J_2_ = 1.5, 1 H), 6.14 (dd, J_1_ = 17.3, J_2_ = 10.4, 1 H), 5.85 (dd, J_1_ = 10.4, J_2_ = 1.5, 1 H), 4.39 (t, J = 6.2, 2 H), 4.18 + 4.17 (t, J = 6.2, 2 H), 4.06 + 4.05 (t, J = 6.5, 2 H), 2.24 (s, 3 H), 2.22 (p, J = 6.2, 2 H), 1.83 (p, J = 7.0 Hz, 2 H), 1.48 (m, 2 H), 1.3–1.4 (m, 4 H) and 0.92 (t, J = 7.0, 3 H).

^13^C-NMR (CDCl_3_, 101 MHz, δ in ppm, *: CH or CH_3_, ^#^: CH_2_): 166.23, 165.05, 164.96, 164.66, 164.57, 163.71, 163.66, 163.24, 163.19, 148.55, 148.49, 147.18, 147.11, 132.44*, 132.42*, 132.40*, 132.38*, 131.91, 131.86, 131.10^#^, 128.39*, 124.25*, 124.21*, 123.03*, 123.00*, 122.05, 121.88, 121.59, 121.43, 120.17*, 120.13*, 114.47*, 114.41*, 68.46, 68.45, 64.79, 61.27, 31.67^#^, 29.18^#^, 28.65^#^, 25.78^#^, 22.71^#^, 16.56*, 14.16 * .

Synthesis of 4-(4-hexyloxybenzoyloxy)-3-methylphenyl 4-(3-acryloyloxypropyloxy)benzoate (**1**) 4.13 g (20 mol) of N,N’-dicyclohexyl carbodiimide was added in portions to a solution of 4.44 g of 4-(hexyloxy)benzoic acid (**7**, 0.16 mol), 7.12 g of 4-hydroxy-3-methylphenyl 4-(3-(acryloyloxy)propoxy)benzoate (**11**, 20 mmol) and 0.25 g (2.0 mmol) of 4-N,N-dimethylaminopyridine in 50 ml of dichloromethane, stirred in an ice bath under a nitrogen atmosphere. After stirring for another 16 h at room temperature, the mixture was filtered through a silica pad and evaporated. About 9.8 g (87% yield) of compound **1** was obtained as a white powder after crystallisation from ethanol and drying over silica in a vacuum desiccator. mp = 78°C and cp = 152°C.

^1^H-NMR (CDCl_3_, 400 MHz, δ in ppm, J in Hz): 8.16 (d, J = 8.8, 2 H), 8.15 (d, J = 8.8, 2 H), 7.18 (d, J = 8.6, 1 H), 7.13 (d, J = 2.7, 1 H), 7.08 (dd, J_1_ = 8.6, J_2_ = 2.7, 1 H), 6.98 (d, J = 8.8, 4 H), 6.43 (dd, J_1_ = 17.3, J_2_ = 1.5, 1 H), 6.14 (dd, J_1_ = 17.3, J_2_ = 10.4, 1 H), 5.85 (dd, J_1_ = 10.4, J_2_ = 1.5, 1 H), 4.39 (t, J = 6.2, 2 H), 4.16 (t, J = 6.2, 2 H), 4.05 (t, J = 6.5, 2 H), 2.24 (s, 3 H), 2.22 (p, J = 6.2, 2 H), 1.83 (p, J = 7.0 Hz, 2 H), 1.59 (water), 1.48 (m, 2 H), 1.3–1.4 (m, 4 H) 0.92 (t, J = 7.0, 3 H).

^13^C-NMR (CDCl_3_, 101 MHz, δ in ppm, *: CH or CH_3_, ^#^: CH_2_): 166.25, 164.97, 164.67, 163.72, 163.20, 148.50, 147.19, 132.44*, 132.42*, 131.92, 131.12^#^, 128.41*, 124.22*, 123.05*, 122.07, 121.45, 120.15*, 114.49*, 114.41*, 68.48^#^, 64.79^#^, 61.30^#^, 31.68^#^, 29.20^#^, 28.67^#^, 25.80^#^, 22.73^#^, 16.58*, 14.17 * .

MALDI-TOF: [M + Na]^+^ calculated for C_33_H_36_O_8_Na:583.23; found: 583.23.

Synthesis of 4-(4-hexyloxybenzoyloxy)-2-methylphenyl 4-(3-acryloyloxypropyloxy)benzoate (**2**) 4.13 g (20 mmol) of N,N’-dicyclohexyl carbodiimide was added in portions to a mixture of 5.0 g of 4-(3-acryloyloxypropyloxy) benzoic acid (**10**, 20 mmol), 6.56 g of 4-hydroxy-3-methylphenyl 4-(hexyloxy)benzoate (**12**, 20 mmol) and 0.24 g (2 mmol) of 4-N,N-dimethylaminopyridine in 50 ml of dichloromethane, stirred in an ice bath under a nitrogen atmosphere. After stirring for another 16 h at room temperature, the mixture was filtered through a silica pad and evaporated. About 5.7 g (70% yield) of compound **2** was obtained as a white powder after crystallisation from ethanol and drying over silica in a vacuum desiccator. mp = 68°C and cp = 154°C.

^1^H-NMR (CDCl_3_, 400 MHz, δ in ppm, J in Hz): 8.17 (d, J = 8.8, 2 H), 8.13 (d, J = 8.8, 2 H), 7.18 (d, J = 8.6, 1 H), 7.13 (d, J = 2.7, 1 H), 7.08 (dd, J_1_ = 8.6, J_2_ = 2.7, 1 H), 6.99 (d, J = 8.8, 2 H), 6.97 (d, J = 8.8, 2 H), 6.43 (dd, J_1_ = 17.3, J_2_ = 1.5, 1 H), 6.14 (dd, J_1_ = 17.3, J_2_ = 10.4, 1 H), 5.85 (dd, J_1_ = 10.4, J_2_ = 1.5, 1 H), 4.39 (t, J = 6.2, 2 H), 4.17 (t, J = 6.2, 2 H), 4.05 (t, J = 6.5, 2 H), 2.24 (s, 3 H), 2.22 (p, J = 6.2, 2 H), 1.83 (p, J = 7.0 Hz, 2 H), 1.48 (m, 2 H), 1.3–1.4 (m, 4 H) and 0.92 (t, J = 7.0, 3 H).

^13^C-NMR (CDCl_3_, 101 MHz, δ in ppm, *: CH or CH_3_, ^#^: CH_2_): 166.13, 164.95, 164.47, 163.57, 163.14, 148.46, 147.01, 132.34*, 132.29*, 131.76, 131.01^#^, 128.29*, 124.15*, 122.90*, 121.78, 121.50, 120.08*, 114.37*, 114.32*, 68.35^#^, 64.70^#^, 61.17^#^, 31.58^#^, 29.09^#^, 28.55^#^, 25.69^#^, 22.62^#^, 16.46*, 14.06 * .

MALDI-TOF: [M + Na]^+^ calculated for C_33_H_36_O_8_Na: 583.23; found: 583.23.

## Synthesis of the CLC oligomers

All oligomeric mixtures are produced following the same general procedure. All solid compounds were added to a small brown glass vial. A small stirring bar was added to each vial. Quantities are chosen such that the molecular ratio of acrylate:thiol is 1:1 for all mixtures. All mixtures contain a 6 wt% chiral monomer **5**. The remainder of the (di)acrylate is a varying mixture of **1**, **2**, **3** and **4**; the relative amounts are noted in Table 1. The total weight of material is approximately 0.5 g for every synthesis.

**Table 1.** Characterisation data of the synthesised oligomers.

Oligomer	LC diacrylate (wt%)^[a]^	Endcapping molecule (wt%)^[a]^	DP^[b]^	PDI^[c]^	T_g_ (°C)^[d]^	T_*i*_ (°C)^[d]^	λ_RT_ (nm)	λ_80_ (nm)
o3.1	4 (32)	1 (51)	2.9	1.48	−26	78	810	660
o3.2	4 (32)	2 (51)	2.8	1.49	−24	81	720	600
o3.3	4 (32)	3 (51)	2.9	1.51	−22	89	850	690
o4.1	4 (46)	1 (37)	3.8	1.67	−23	78	860	630
o4.2	4 (46)	2 (37)	3.7	1.69	−24	76	780	600
o4.3	4 (46)	3 (37)	3.7	1.69	−21	84	850	630

DP - Degree of polymerisation; PDI – Poly Dispersity Index; T_i_ – Isotropic transition temperature; T_g_ – Glass transition temperature; λ_RT_ – peak reflective wavelength at room temperature; λ80 – peak reflective wavelength at 80°C. [a] The remaining weight percentage is DODT and chiral dopant 5 [b] Determined by ^1^H-NMR. [c] Determined by GPC. [d] Determined by DSC.

In a separate vial, the appropriate amount of DODT is added using a Finn-pipet. The DODT is then dissolved into 2 ml of DCM and added to the reaction vial. An additional 1 ml of DCM is used to wash out the remainder of the DODT vial and is added to the reaction vial. Ten microlitre of di-n-propylamine is then added to the reaction mixture as a catalyst. The vials are shut tight with a lid and placed on a hotplate at 35°C. The stirring is activated at 200 rpm, and the reaction is left overnight. Samples of 100 μl are taken to perform ^1^H-NMR and GPC measurements. Oligomers were dried by leaving them at 50°C overnight to evaporate excess solvent.

## Preparation of the CLC oligomer coatings

Coatings are produced by dissolving the oligomer mixtures in cyclopentanone (50 wt%). To this mixture is added 5 wt% (relative to the dry oligomer) of a 1 wt% Byk-361N solution in cyclopentanone. Stirring bars are added to the vials, after which they are closed with a lid and heated to 80°C while stirring for 15–30 min until the viscous reaction product fully dissolves. The inks are applied to the non-treated side of a 50 μm thick, 5-cm-wide substrate via the wire bar coating technique using an RK-K control coater. Both sides of a strip of PET are rinsed with isopropanol, then dried with compressed nitrogen. The top edge of a strip is taped to the top end of the bar-coater table. A wire bar with a 12 μm gap is placed in the holder and firmly pressed onto the strip. The oligomer mixture (350 μl) is carefully applied at the wire bar/PET interface, ensuring that the whole width of the strip is covered. The machine is then turned on at a speed of approximately 1 cm/s. Resulting coatings are placed in the oven for 1 h at 60°C to evaporate the remaining solvent. This process is repeated for all oligomers.

## Temperature-dependent transmission experiments

All UV–Vis data were collected within 24 h of production of the coating in question. Transmission spectra are measured on a small strip cut from the coated foils with air as a baseline. The baseline is taken with the heater module (Linkam LNP-96S) mounted in the machine (PerkinElmer LAMBDA 750 UV/Vis/NIR spectrophotometer). After the baseline is taken, a small strip is cut from the coated foil and clamped onto the heating module. The stage is set to 20°C, and a measurement is taken. Temperatures shown in the spectra are the set temperatures of the hot stage. After completion of the measurement, the coating is heated at a variable rate. Unless mentioned otherwise, the coating is heated by 10°C at a rate of 5°C/min, then left to rest at that temperature for 2 min. The next measurement is then taken, and this cycle is repeated until the measurement no longer shows a reflection band. The coatings are then cooled down at a rate of 10°C/hour. Every half hour, a measurement is taken until the coating reaches its resting temperature of 20°C or 30°C as shown per measurement. The temperatures reported for every measurement correspond to the setting of the Linkam hot stage.

## Results

### Preparation of the monoacrylate mesogens

Three different monoacrylate mesogens are synthetised for preparing the endcapped cholesteric LC oligomers. They are prepared via comparable synthetic routes as outlined in Scheme 1. Monoacrylates **1** and **2** are prepared in their isomerically pure forms in a six-step synthesis, while **3** is a one-to-one mixture of **1** and **2** prepared in four steps. Monoacrylate **3** is synthesised by making use of isomeric mixture **6**. The isomerically pure constituents of **6**, prepared via two additional reaction steps [39], are used for the synthesis of **1** and **2** (Figure S2). By making use of the Steglich esterification reaction, either benzoic acid derivative **7** or **10** is added to the isomeric core. The protecting THP-ether is then removed, and the other acid is reacted with the deprotected products **9**, **11**, and **12** to form the final monoacrylate compounds. The detailed synthesis route and the characterisation data are provided in the experimental section and supporting information (Figure S3-S5). Of particular note is the thermal analysis data of the different compounds, showing crystalline nematic transitions at 78°C, 68°C, and 67°C for **1**, **2**, and **3,** respectively. The nematic to isotropic transition occurs at 153°C for all three compounds.

### Synthesis of cholesteric liquid crystal oligomers

**Figure 1.**
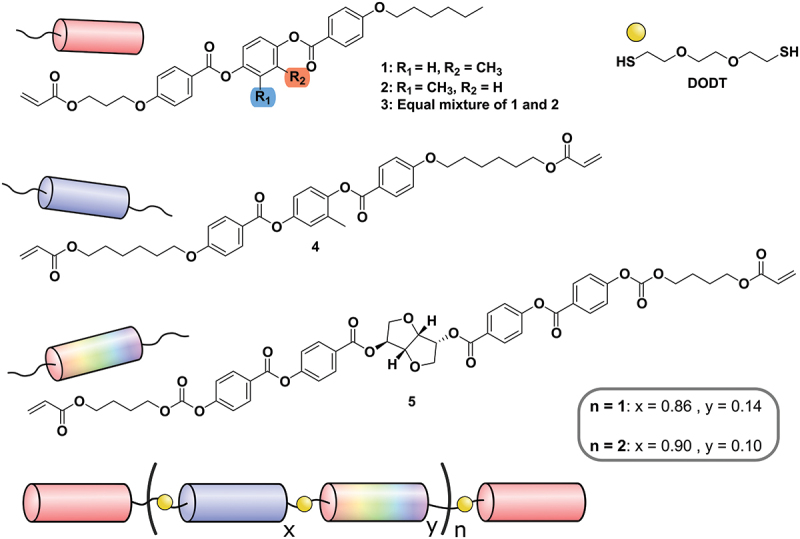
(Colour online) Schematic representation of the synthesised oligomers.

To study the effect of the different structural isomers of the monoacrylate mesogens on the thermochromic coatings, different oligomers were synthesised (Figure 1). The oligomers are designed with an average degree of polymerisation (DP) of 3 or 4. The DP refers to the average number of mesogens per chain, counting both the mono- and diacrylates. For both DPs, oligomers are synthesised with one of the monoacrylates **1** through **3**. The oligomer with a DP of 3 and endcapped with **1** is named **o3.1** and so on (Table 1). All mixtures were synthesised using one of the monoacrylate mesogens **1** through **3**, diacrylate mesogen **4**, dithiol chain extender DODT, and supplemented with 6 wt% chiral dopant **5**, resulting in coatings that reflect in the NIR-spectrum at room temperature (vide infra). All the reagents are dissolved in dichloromethane (DCM), and 1 wt% of the basic catalyst was added. After the synthesis, the solvent is evaporated, and the oligomer is used without further workup. The final result is six different oligomers, where the only difference between oligomers with the same DP is the endcapping molecule used. The oligomers were characterised by nuclear magnetic resonance (NMR), gel permeation chromatography (GPC) and differential scanning calorimetry (DSC) measurements. Key results from these measurements as well as the compositions of all oligomers are summarised in Table 1. The spectra are available in the supporting information (Figure S6-S7 and Table S1).

By comparing characteristic ^1^H-NMR peaks of aromatic groups present in the CLC mixture and comparing them to remaining acrylate signals, it is found 93% or more of acrylate groups are consumed in all mixtures, indicating a near-complete conversion. By making use of the relative intensities of peaks characteristic of the mesogens and the thiol spacer, and correcting for the achieved conversion, the DP can be determined for every mixture. These values are all near the expected value based on the reaction feed (Table 1).

**Figure 2.**
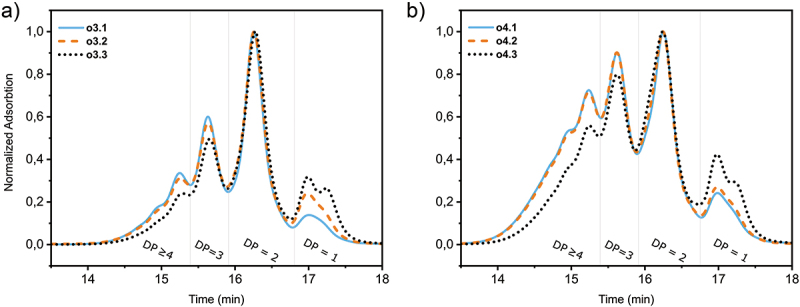
(Colour online) Overlayed normalized GPC-traces of all six oligomer mixtures. a) GPC traces of mixtures with a DP of 3. b) GPC traces of mixtures with a DP of 4. All spectra are normalized to their DP = 2 peak.

In GPC, the average molecular weight of the different oligomer lengths of around 2000 to 3000 g/mol results in distinct observable peaks for the various oligomer lengths (Figure 2). Several identifiable peaks appear. The peak with the longest retention time corresponds to unreacted monomers. Peaks at decreasing retention times correspond to progressively longer oligomers. As expected, the synthesised tetramers (**o4**) contain relatively more long oligomer chains than the trimers (**o3**). For both the trimers and tetramers, the traces of the oligomers with different endcapping molecules overlap closely, expressed in near-identical polydispersity indices (PDIs). It can also be seen that all three endcapping molecules give identical retention times for all peaks for all the different oligomeric isomers.

The oligomers containing monoacrylates **1** and **2** have very similar isotropic transition temperatures, around 80°C (Figure S7). The oligomers with **3** have slightly elevated isotropic transition temperatures, which is the opposite response found in the monomeric materials. None of the DSC-traces show any transition peaks besides their glass transition and cholesteric-isotropic transition.

### Structurally coloured NIR reflective coatings

**Figure 3.**
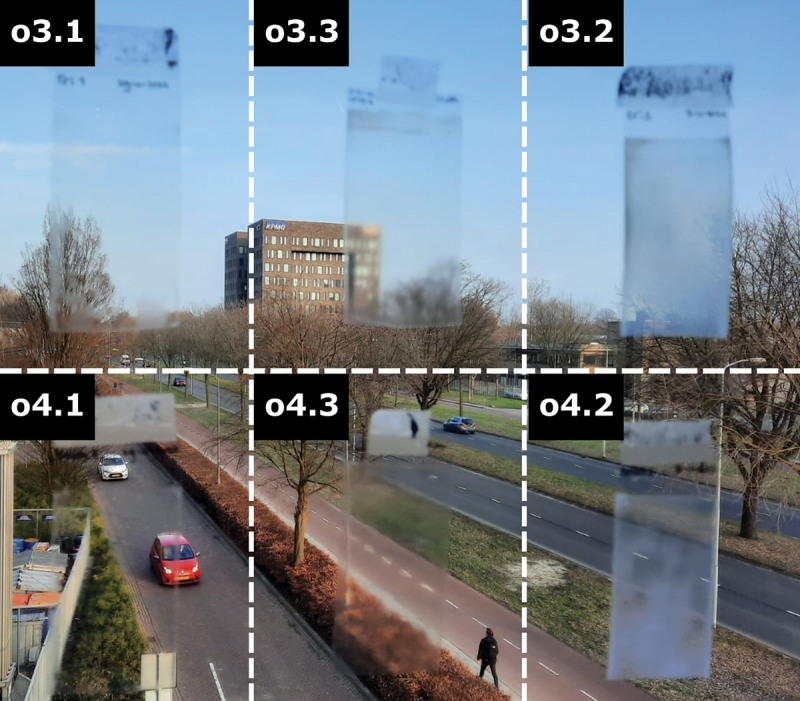
(Colour online) The different oligomer coatings taped to a window. The top left corner indicates which oligomer coating is in its respective space.

To prepare the structurally coloured coatings, the six oligomers are individually dissolved in cyclopentanone and applied to a piece of polyethylene terephthalate (PET) foil via wire bar coating at room temperature. The coatings are placed in the oven at 60°C for 1 h to evaporate the solvent and allow them to align into the cholesteric phase. The coatings are then left to rest at room temperature overnight and taped to a window to compare their visual properties (Figure 3).

Optically, the coatings of the tri- and tetramers using the same endcapping molecules appear mostly identical. However, there are significant differences between the oligomers with different endcapping molecules. Both oligomers endcapped with **1** (**o3.1**, **o4.1**) are fully transparent, only showing some edge defects resulting from the coating procedure. Those endcapped with **2** (**o3.2**, **o4.2**), on the other hand, show significant haziness to the point that they are almost opaque. This behaviour was observed immediately after coating and did not change over time [40]. It appears that the different isomers of the endcapping molecules result in significantly different aligning properties of the oligomeric coatings, despite their similar chemical compositions and phase transition temperatures. This is additionally shown by the coatings of the mixtures with endcap **3** (**o3.3**, **o4.3**), which are more scattering than those of **1** but much more transparent than those with **2**. As **3** is simply a one-to-one mixture of the isomers **1** and **2**, it appears that its optical properties are likewise an intermediate between the two isomerically pure oligomers.

### Thermochromic response of the structurally coloured NIR reflective coatings

**Figure 4.**
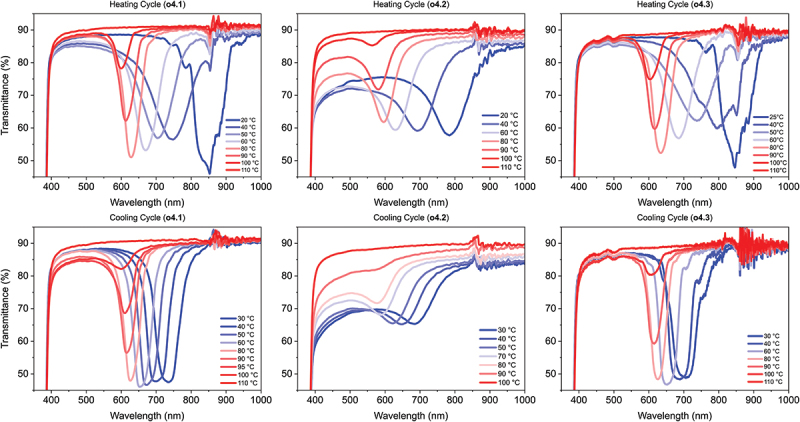
(Colour online) Temperature dependent transmission data taken during the heating (5 °C/min, top row) and cooling (10 °C/hour, bottom row) cycles of the tetramer coatings. Artifacts at 860 nm are the result of the detector switch from NIR to visible light.

Both the coatings prepared with tetramers and trimers show a thermochromic response (Figure 4 and S8, respectively). The position of the reflection band at room temperature and at 80°C are noted in Table 1. It can be clearly observed that in all cases, the oligomers are in a cholesteric state at room temperature reflecting wavelengths around 800 nm, in the NIR region. The room-temperature reflection bands of **o4.1** and **o4.3** are redshifted approximately 50 nm compared to **o4.2**, which could be explained by the observed difference in alignment or by a slight experimental deviation in the chiral dopant concentration. While the reflection bands of **o4.1** and **o4.3** are sharp and the transmissions in the visible region are above 80%, the room-temperature transmission peak of **o4.2** is more broad and the transmission is around 70% in the visible region. This behaviour of the transmission is indicative of scattering as also observed earlier (Figure 3).

All coatings display a blueshift of their reflection bands upon heating, as previously demonstrated for endcapped oligomers with a similar composition [36]. This hypsochromic shift of the coatings is broadly similar in terms of initial and final reflected wavelengths, with **o4.2** slightly blueshifted relative to **o4.1** and **o4.3** [41]. For **o4.2**, the heating cycle does not appear to positively affect its alignment. Throughout the cycle, it maintains its relatively shallow reflection peak and scattering properties while still obviously blueshifting. When the coatings are heated to above their isotropic states they become fully transparent, reaching 90% transmission due to absorption from the PET-foil itself. When cooling the coatings from the isotropic state, they recover with the same characteristics they displayed before they were isotropic – **o4.1** and **o4.3** recover their narrow, deep reflection bands almost immediately, and **o4.2** maintains its broader reflection band and scattering properties in the visible spectrum. This recovery from the unaligned isotropic transparent state demonstrates that the degree of scattering and alignment is inherent to the chemical composition of the oligomers. It can also be clearly observed that for all coatings, the reflected wavelength at room temperature does not fully recover to its initial position. Instead, as the cooling cycle progresses, the peaks are significantly blue shifted relative to their heating cycle position at the same temperatures.

When comparing the positions of the reflection bands during the heating and cooling cycles, some notable differences can be observed (Figure 4). As mentioned, the position of the reflection band appears to be delayed compared to the heating cycle, particularly at temperatures near room temperature. In addition, while **o4.1** and **o4.3** maintain sharp reflection peaks throughout the entire cooling cycle, during heating until 60°C the bands broaden and become slightly less deep. This indicated a slight decrease in alignment, which is recovered at higher temperatures. These results imply that for temperatures below 50°C, the coatings may experience kinetic constraints on their response time due to the viscosity of the material, working against the thermodynamic driving force for the phase change.

Notably, the reflected wavelength shift increases when comparing the trimer to the tetramer of all endcapped oligomers, regardless of endcapping isomer (Table 1). In the best case for trimers, the hypsochromic shift between room temperature and 80°C is around 170 nm, while in case of tetramer, this is around 230 nm. This is likely the result of the ratio of hexyl to propyl spacers of the mesogenic units: it was previously demonstrated in a similar system that increasing the relative amount of hexyl- to propyl-spacers results in an increased pre-transitional effect and thus in a more significant thermal response [34,36]. As only the endcapping molecules contain propyl spacers, higher DP mixtures contain relatively more hexyl spacers and experience a more significant shift. In this case, it appears that the effect of the spacer lengths can overcome the decreased ability to align molecules that can be expected in the higher DP and more viscous mixtures.

**Figure 5.**
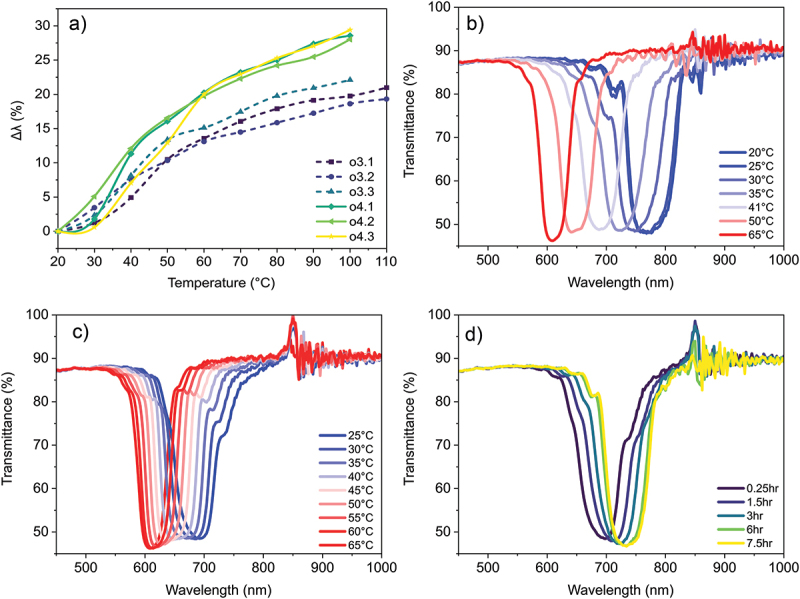
(Colour online) Expanded look into the thermal response of oligomers. a) Relative shift of the reflection peak for all oligomers upon heating, with respect to the starting position, for the trimeric (dashed lines) and tetrameric (solid lines) oligomer coatings. The wavelength shift Δλ is calculated as (λ(RT) - λ(T))/λ(RT) * 100%. b) Temperature-dependent transmission data of o4.1 at 6 °C/hour. c) Continuation of the same temperature cycle, cooling the coating at 10 °C/hour. d) Continuation of the same temperature cycle, maintaining the coating at 25°C.

As the starting wavelengths are not the same across different oligomers, it is difficult to compare them directly. Instead, they may be compared using a normalised shift, calculated by taking the percentage change of the reflection band position with respect to the position at room temperature (Figure 5a). The normalised graphs show clearly that the specific monoacrylate isomers have a minimal effect on the overall spectral shift; all three trimers and all three tetramers follow roughly the same trend, exhibiting the same thermochromic behaviour. This implies that the different aligning properties are independent from the pre-transitional effect experienced by the oligomer and consequently that the oligomers have similar smectic-to-cholesteric phase transition temperatures; this behaviour is also exhibited by comparable endcapped oligomers (Figure S9) [36].

To characterise the effect of the temperature gradient and the kinetic limitations of the response further, **o4.1** was reproduced. The coating of this mixture was then subjected to a temperature gradient of 6°C per hour for the heating cycle and 10°C per hour for the cooling cycle, mimicking environmental condition the coating can be subjected to when applied as a window film. The coating is first heated to 65°C, then cooled to 25°C, and finally held at that temperature overnight (Figures 5b-d). Note that all peaks are slightly blueshifted compared to the original coatings because of a slightly increased chiral dopant concentration. There are notable differences between the series shown in Figure 5 compared to the experiments in Figure 4. During heating, broadening of the reflection band does not occur. Instead, it remains well defined at all temperatures and remains at the 50% reflection limit throughout. This indicates that the slight decrease in the quality of the reflection bands in Figure 4. is the result of the phase transition within the material being kinetically limited and that with this lower temperature gradient, the shift is more thermodynamically controlled. Some kinetic limitations are still observed. No change occurs between 20°C and 25°C, indicating that change around room temperature is still limited kinetically at this temperature gradient. Secondly, as was the case previously, the cooling cycle lags when comparing the reflection bands at given temperatures. Upon reaching 25°C, the centre reflection band is approximately 50 nm shifted from its eventual resting point or 6.5% with respect to the initial wavelength. Within 3 h it is within 10 nm of equilibrium, which is reached after 6 h. This equilibrium is notably offset from the measurement at 25°C during the heating cycle. Thus, while recovery around room temperature is fairly slow, the response across the whole temperature range keeps up well with this moderate temperature gradient.

## Conclusion

We have successfully demonstrated the impact of different isomers of monoacrylate endcapping molecules on the properties of a thermochromic liquid crystal oligomer film. Synthetic routes were designed to produce two isomers of a monoacrylate endcap molecule as well as their one-to-one mixture. While the synthesis route leading to an isomeric mixture of the monoacrylates removes two steps from the synthetic procedure compared to the isomerically pure compounds, the poor alignment properties that stem from one of the constituent isomers render this undesirable for application purposes. It is thus vital to consider the isomer chosen for the endcapping molecule when working with cholesteric liquid crystal oligomers. It was shown that oligomers prepared with one of the isomerically pure endcapping molecules resulted in coatings with equal or better thermochromic properties as a previously reported endcapping molecule while cutting down the required synthesis steps for the endcap molecule from 9 to 6. The oligomers prepared with the other isomer only produced scattering coatings. All isomers were shown to be able to achieve a shift of their reflective wavelength of approximately 30% with respect to their starting value when incorporated into a tetramer, further illustrating the minute differences between them. The best performing oligomer achieved a blueshift of approximately 250 nanometres between room temperature and its isotropic transition.

The response kinetics of the best performing oligomer were more thoroughly characterised. It was found that at reasonably fast temperature, increase of several degrees per minute the alignment is disturbed at low temperatures, which can be prevented by decreasing the rate to several degrees per hour. When cooling the coatings at a slower rate, the reflected wavelength is unable to achieve the same degree of responsivity as in the heating cycle; the reflective wavelength at a given temperature consistently lags behind compared to the position during the heating cycle. This is likely caused by the increasing viscosity of the oligomer upon decreasing the temperature. This could be resolved by lowering T_g_. By tuning the reflected wavelengths slightly further into the infrared and making use of the excellent blueshifting properties of the material, these oligomer films may find use in thermal regulatory window films.

## Supplementary Material

Supplemental Material
