# Supplementary material for: On the isomeric purity of endcap molecules in cholesteric liquid crystal oligomers for near-infrared thermochromic coatings
Source: Liq Cryst. 2024 May 26;51(10):1651–63. doi: 10.1080/02678292.2024.2350046 (PMC11529603; doi:10.1080/02678292.2024.2350046)
Supplement: Supplemental Material [file TLCT_A_2350046_SM0413.pdf]

Supporting Information

On the Isomeric Purity of Endcap Molecules in Cholesteric Liquid Crystal Oligomers for Near-Infrared Thermochromic Coatings

Henk Sentjens, Janneke M.A. Bloemers, Johan Lub, Carmen Luengo Gonzalez, Augustinus J.J. Kragt, Albert P.H.J. Schenning

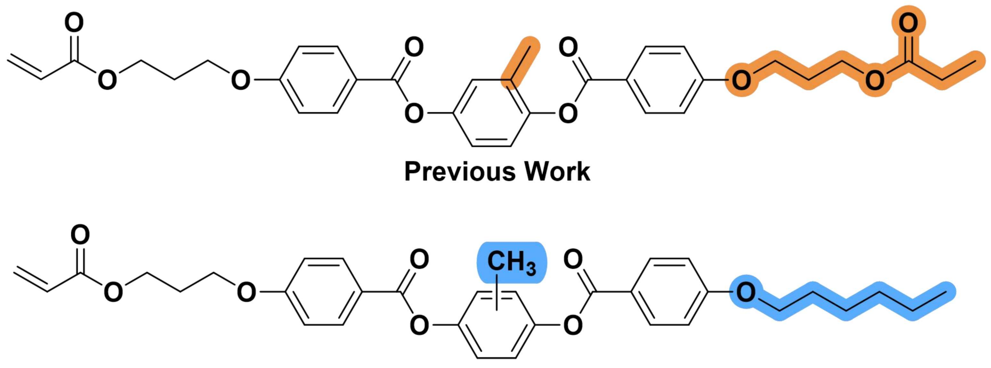

Figure S1. Monoacrylate compound synthesized in previous work[1] and compound synthesized in this work, with key differences indicated in color. The simplification of the monoacrylate in this work is achieved by replacement of the non-reactive alky tail in the molecule and working with mixtures containing different isomers.

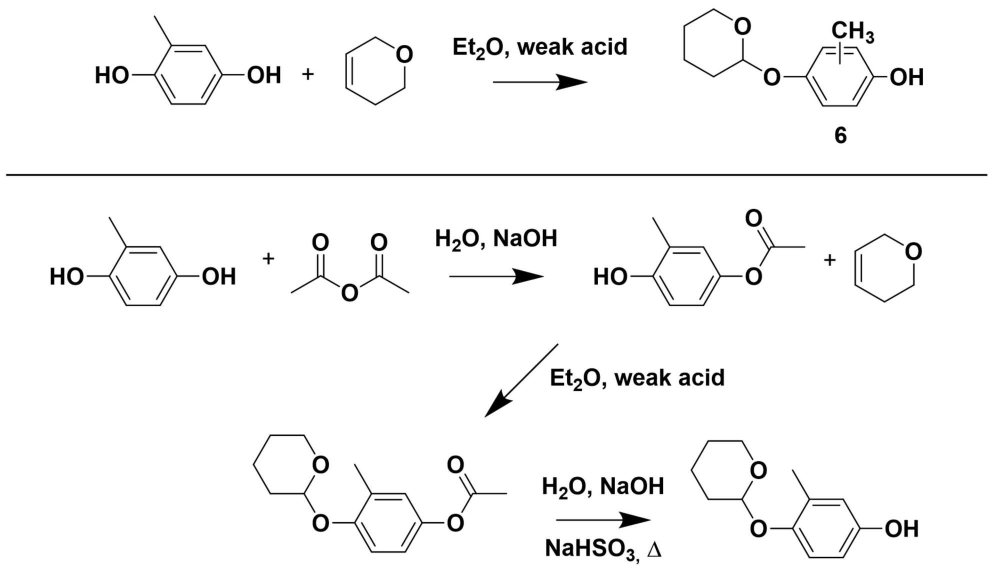

Figure S2. Synthesis of isomeric mixture **6** (top) and its 3-methyl isomer (bottom) as reported previously. [1–4]

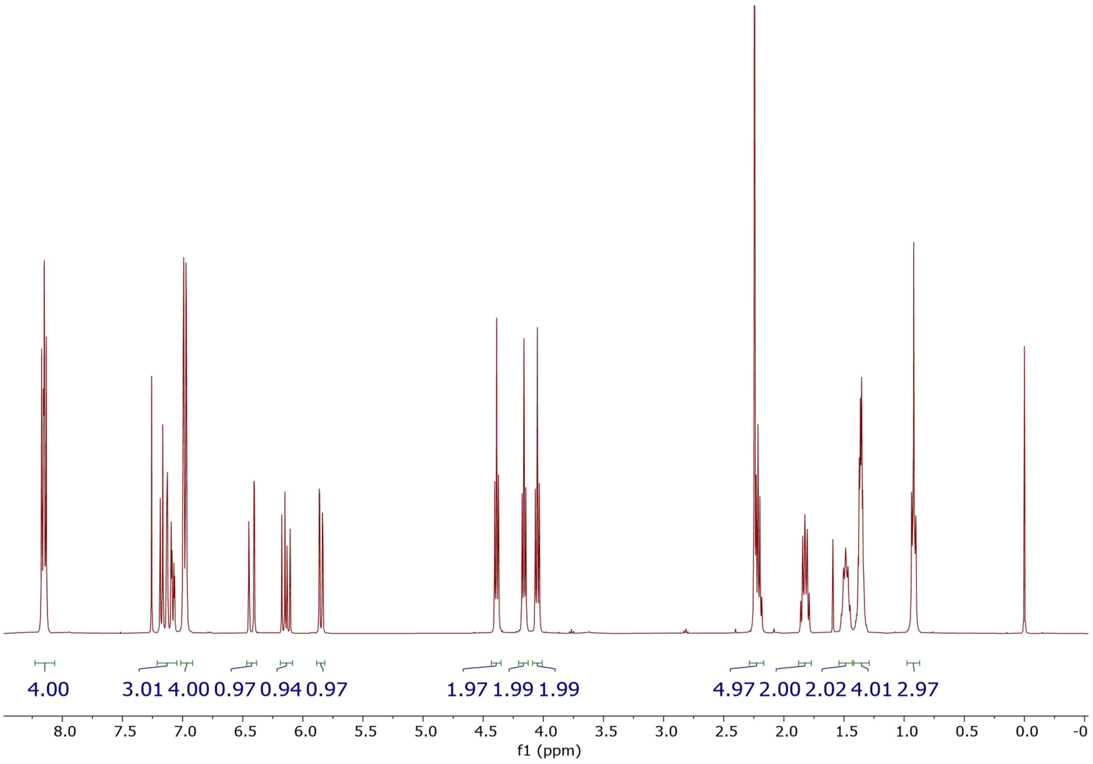

Figure S3a. ^{1}H-NMR spectrum of **1**.

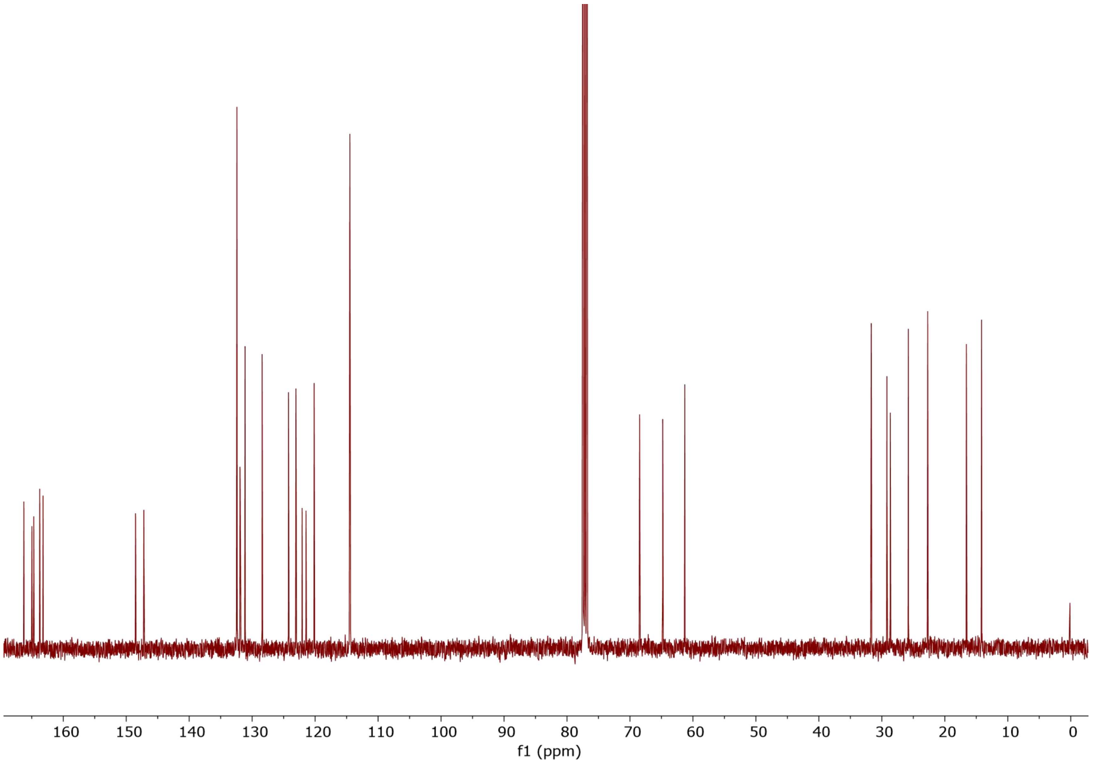

Figure S3b. ^{13}C-NMR spectrum of **1**.

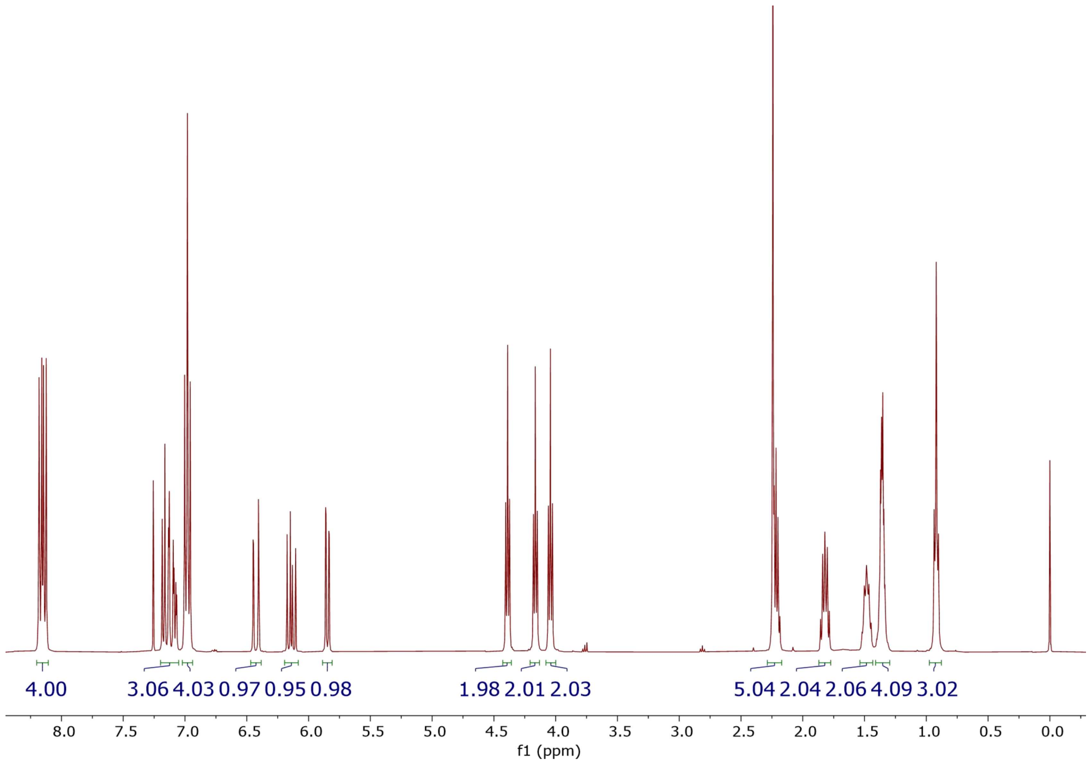

Figure S3c. ^{1}H-NMR spectrum of **2**.

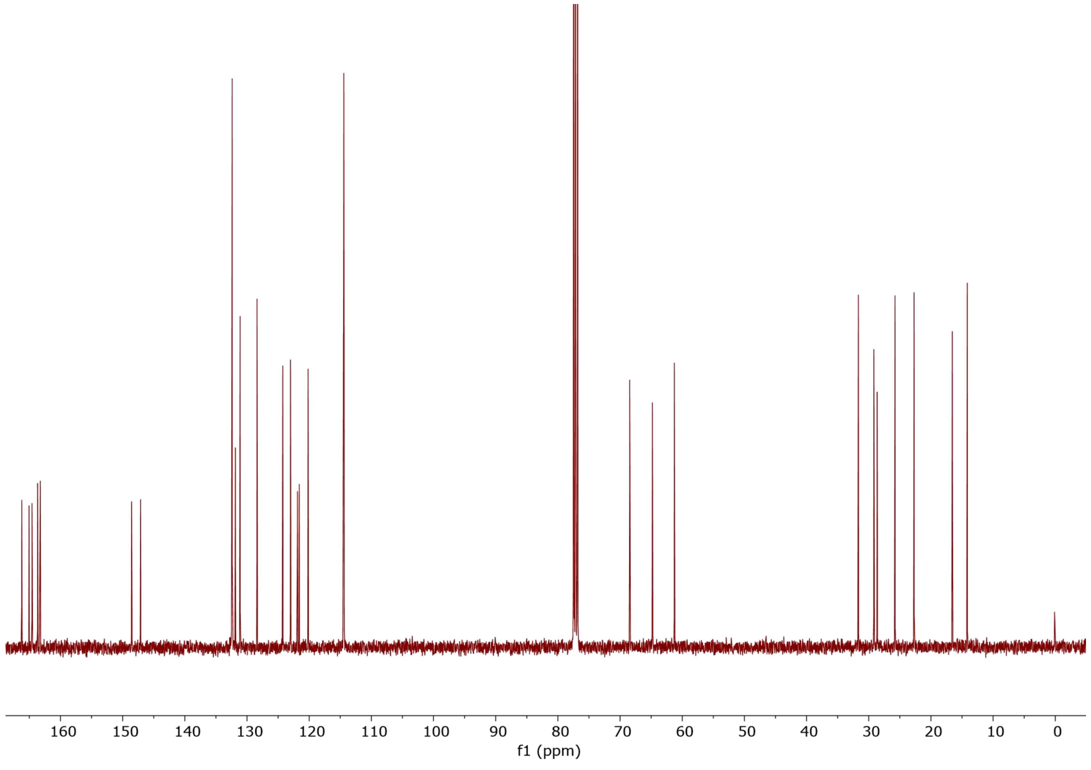

Figure S3d. ^{13}C-NMR spectrum of **2**.

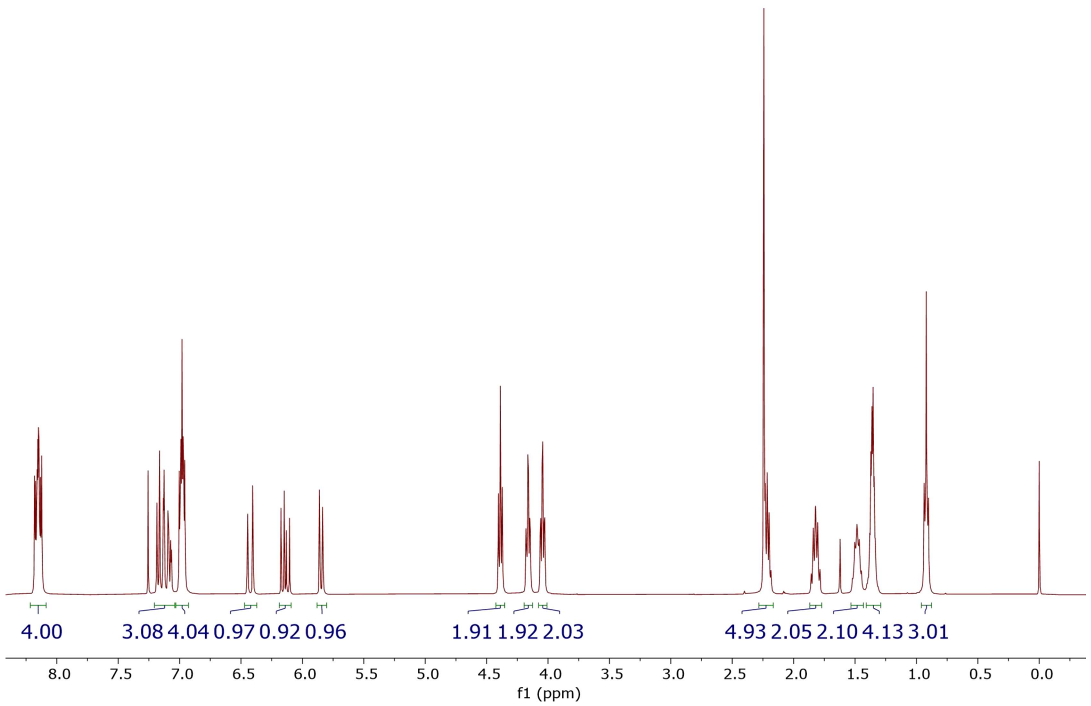

Figure S3e. ^{1}H-NMR spectrum of **3**.

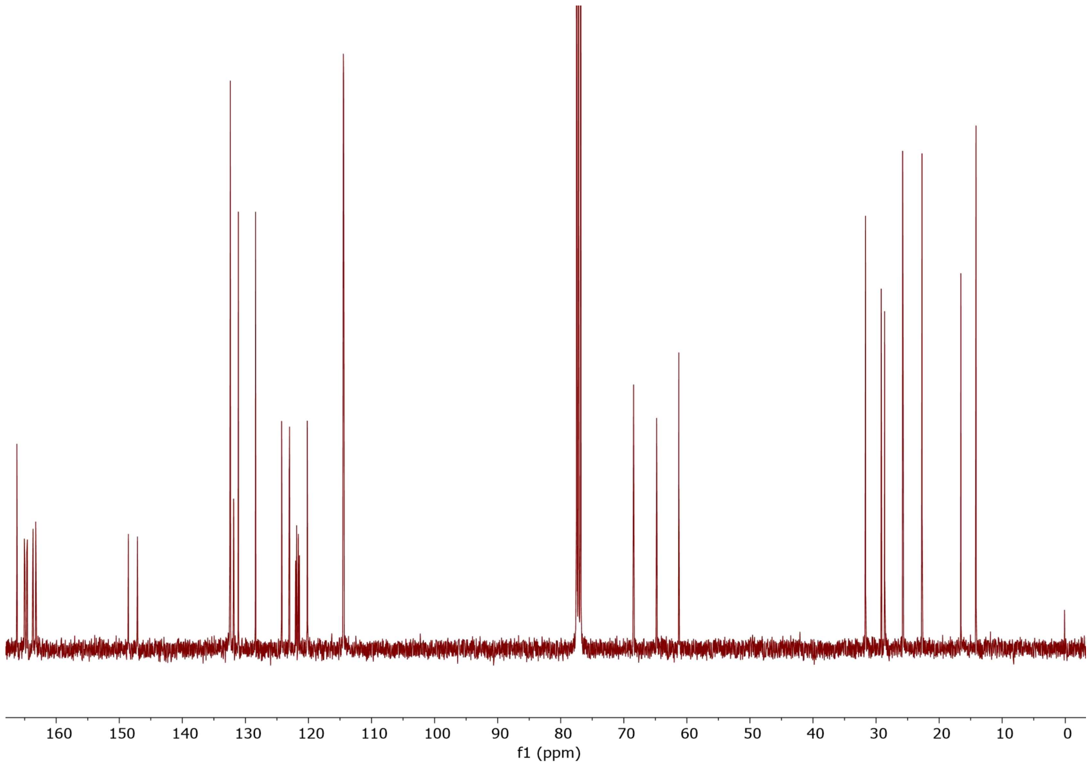

Figure S3f. ^{13}C-NMR spectrum of **3**.

1

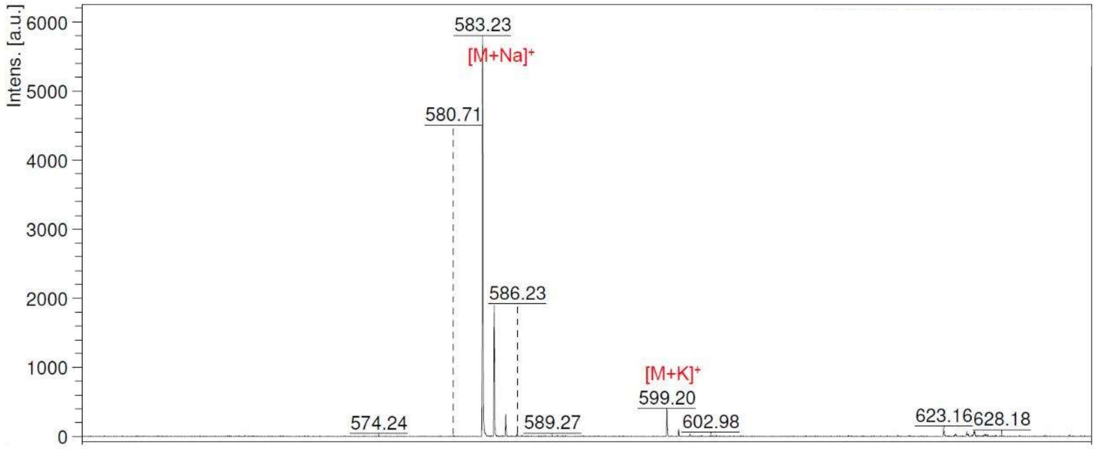
Intens. [a.u.]
583.23
[M+Na]+
580.71
586.23
[M+K]+
599.20
574.24
589.27
602.98
623.16
628.18

2

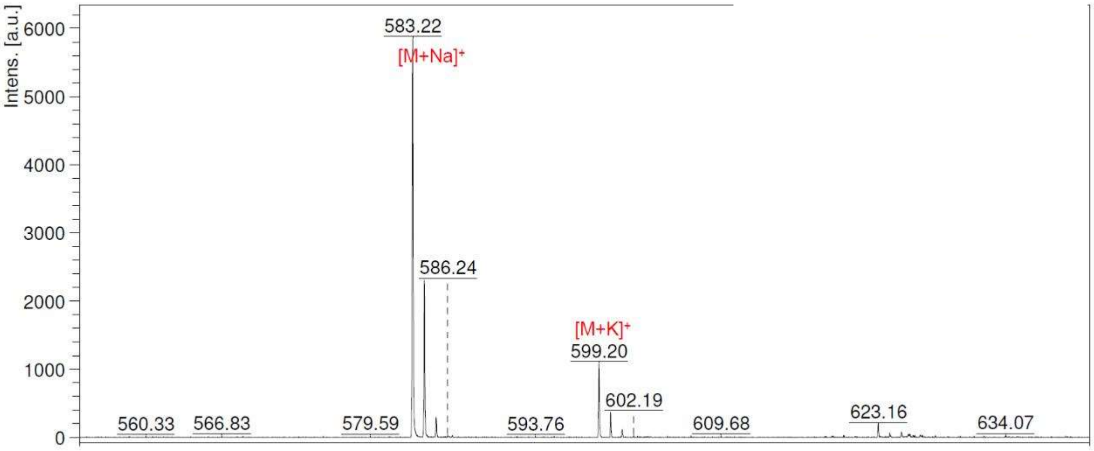
Intens. [a.u.]
583.22
[M+Na]+
586.24
[M+K]+
599.20
602.19
560.33
566.83
579.59
593.76
609.68
623.16
634.07

3

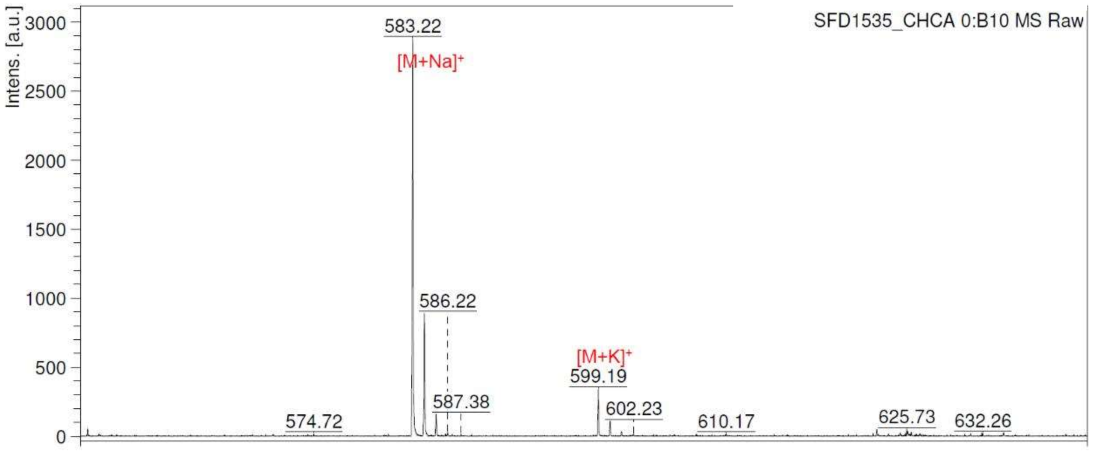
Intens. [a.u.]
SFD1535_CHCA 0:B10 MS Raw
583.22
[M+Na]+
586.22
587.38
[M+K]+
599.19
602.23
574.72
610.17
625.73
632.26

Figure S4. MALDI-TOF MS spectra of **1**, **2**, and **3**.

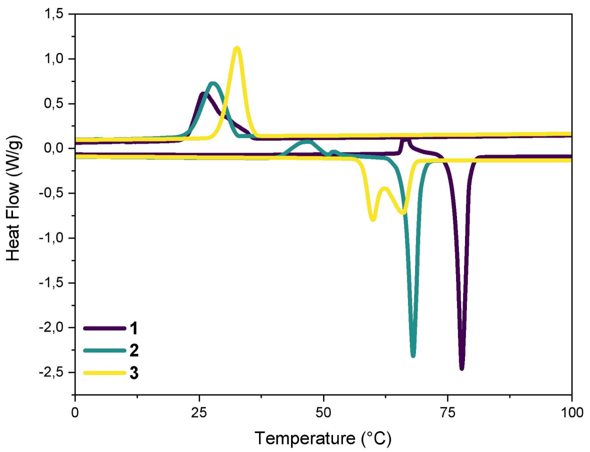

Figure S5. Overlayed DSC curves of **1**, **2**, and **3**. Besides their crystalline-nematic transition temperatures, small upwards peaks can be observed in both **1** (68°C) and **2** (50°C). These likely correspond to recrystallization effects experienced by the pure monomers, which is notably absent in **3**. In turn, it appears to transition twice, likely as a result of the constituent compounds. The latter temperature was taken as its overall crystalline-nematic transition temperature.

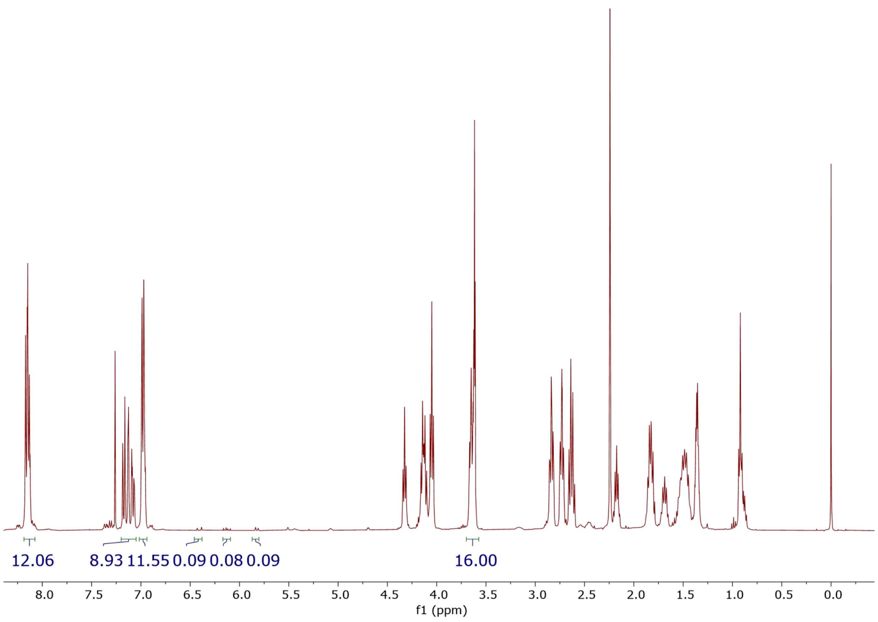

Figure S6a. ^{1}H-NMR spectrum of **o3.1.** For all oligomers, DP is determined by first setting peaks corresponding to the center carbons of DODT (3.65, (**t**, 4H); 3.62 (**s**, 4H)) to 8*(theoretical DP - 1). The aromatic peaks of the mesogen units are then integrated, compared to their theoretical values, and corrected for the conversion of the acrylates. These values are then used to calculate DP. The contribution of the chiral dopant is not considered. DP $= \frac{12.06+8.93+11.55}{33} * \frac{12-(0.09+0.08+0.09)}{12} * 3 = 2.89$.

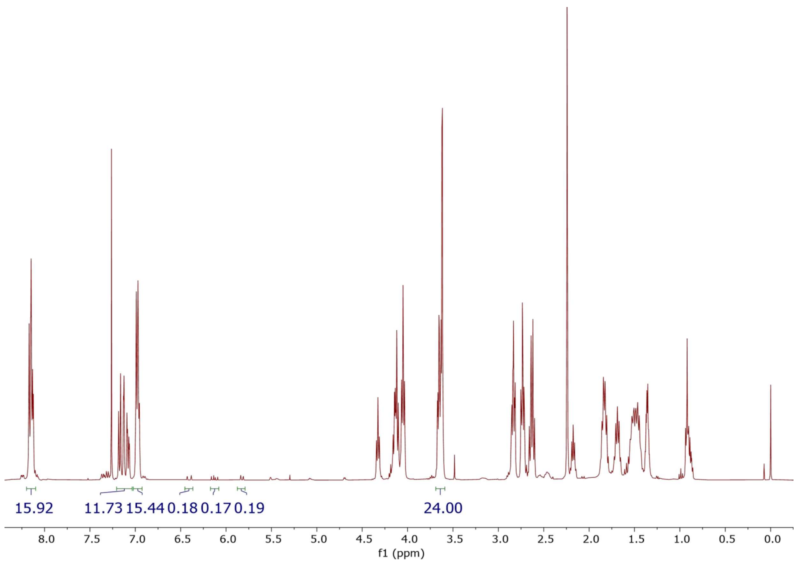

Figure S6b. ^{1}H-NMR spectrum of **o4.1.** DP $= \frac{15.92+11.73+15.44}{44} * \frac{18-(0.18+0.17+0.19)}{18} * 4 = 3.80$.

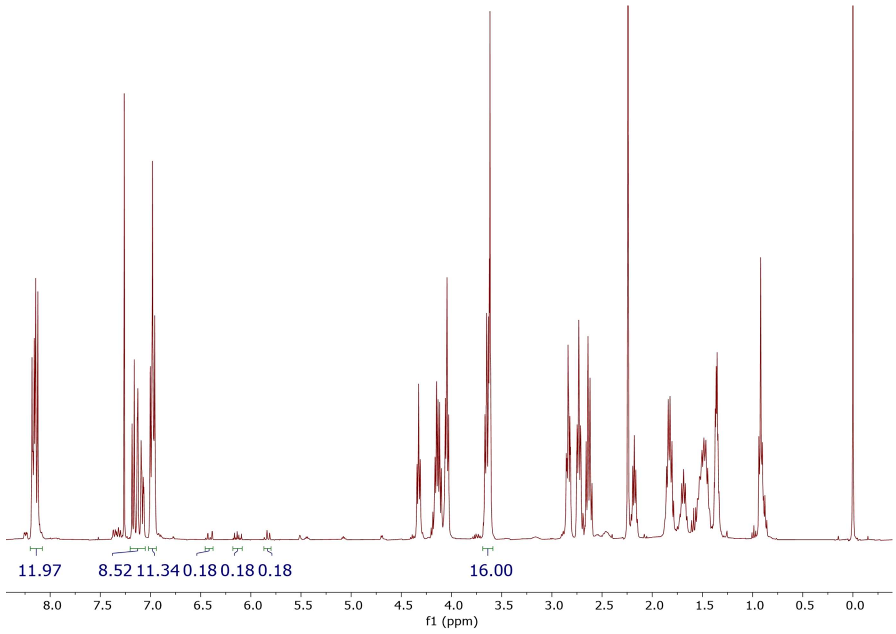

Figure S6c. ^{1}H-NMR spectrum of **o3.2**. DP = $\frac{11.98+8.52+11.34}{33} * \frac{12-(0.18+0.18+0.18)}{12} * 3 = 2.76$.

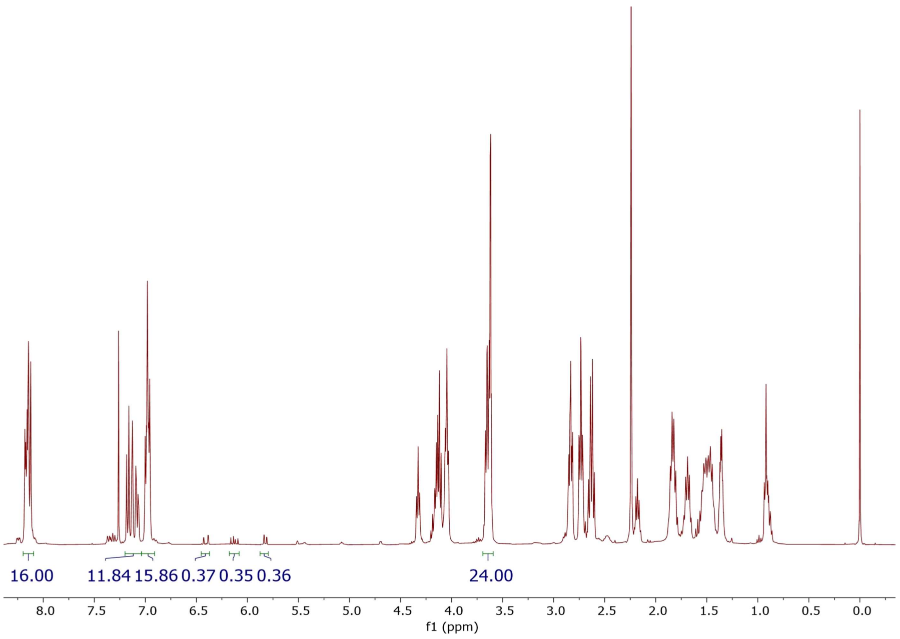

Figure S6d. ^{1}H-NMR spectrum of **o4.2**. DP = $\frac{16+11.84+15.86}{44} * \frac{18-(0.37+0.35+0.36)}{18} * 4 = 3.73$.

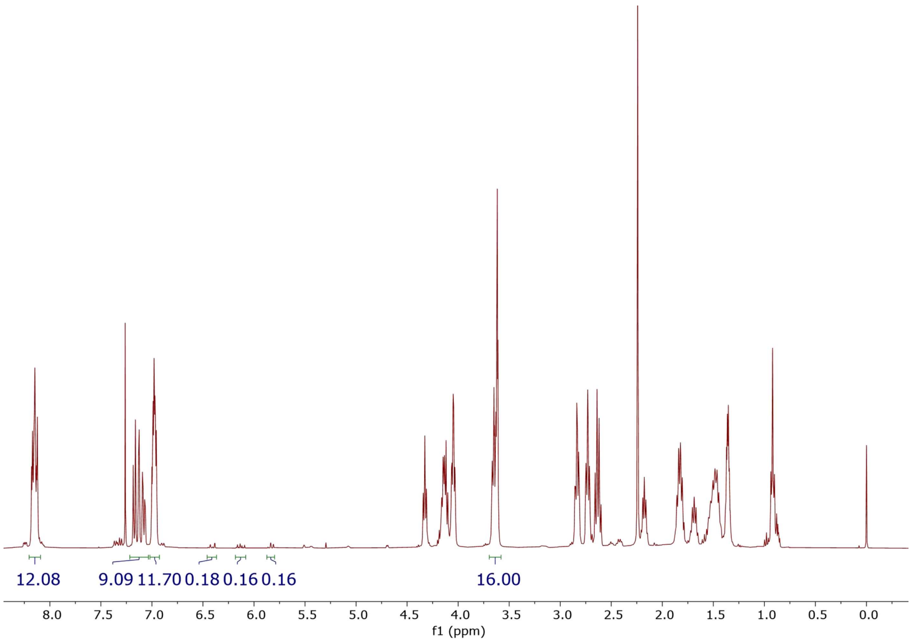

Figure S6e. ^{1}H-NMR spectrum of o3.3. DP = $\frac{12.08+9.09+11.70}{33} * \frac{12-(0.18+0.16+0.16)}{12} * 3 = 2.86$.

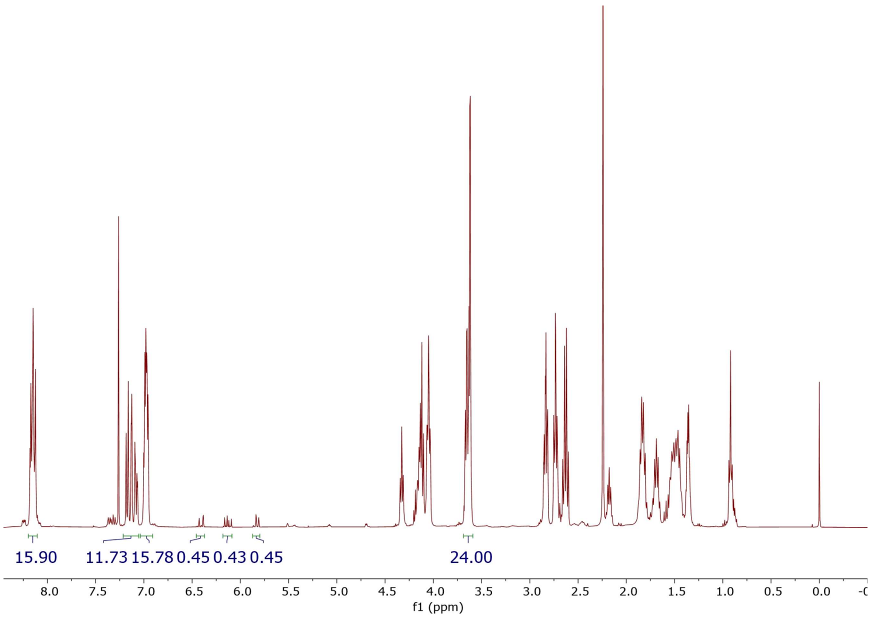

Figure S6f. 1H-NMR spectrum of **o4.3**. DP = $\frac{15.90+11.73+15.78}{44} * \frac{18-(0.45+0.43+0.45)}{18} * 4 = 3.65$.

^{1}H-NMR shows the signals of the individual components of the oligomers, as well as signals characteristic of the sulfur-carbon bond after the chain extension reaction and subsequent

disappearance of the acrylate bond (2.60-2.88 ppm, Figure S6). The conversion of the reaction can be determined by the leftover acrylate in the mixture following the reaction.

Table S1. GPC data of the synthesized oligomers

	M_n (g/mol)	M_w (g/mol)	PDI (-)
o3.1	2404	3560	1.48
o4.1	2823	4705	1.67
o3.2	2187	3262	1.49
o4.2	2744	4643	1.69
o3.3	1867	2827	1.51
o4.3	2235	3778	1.69

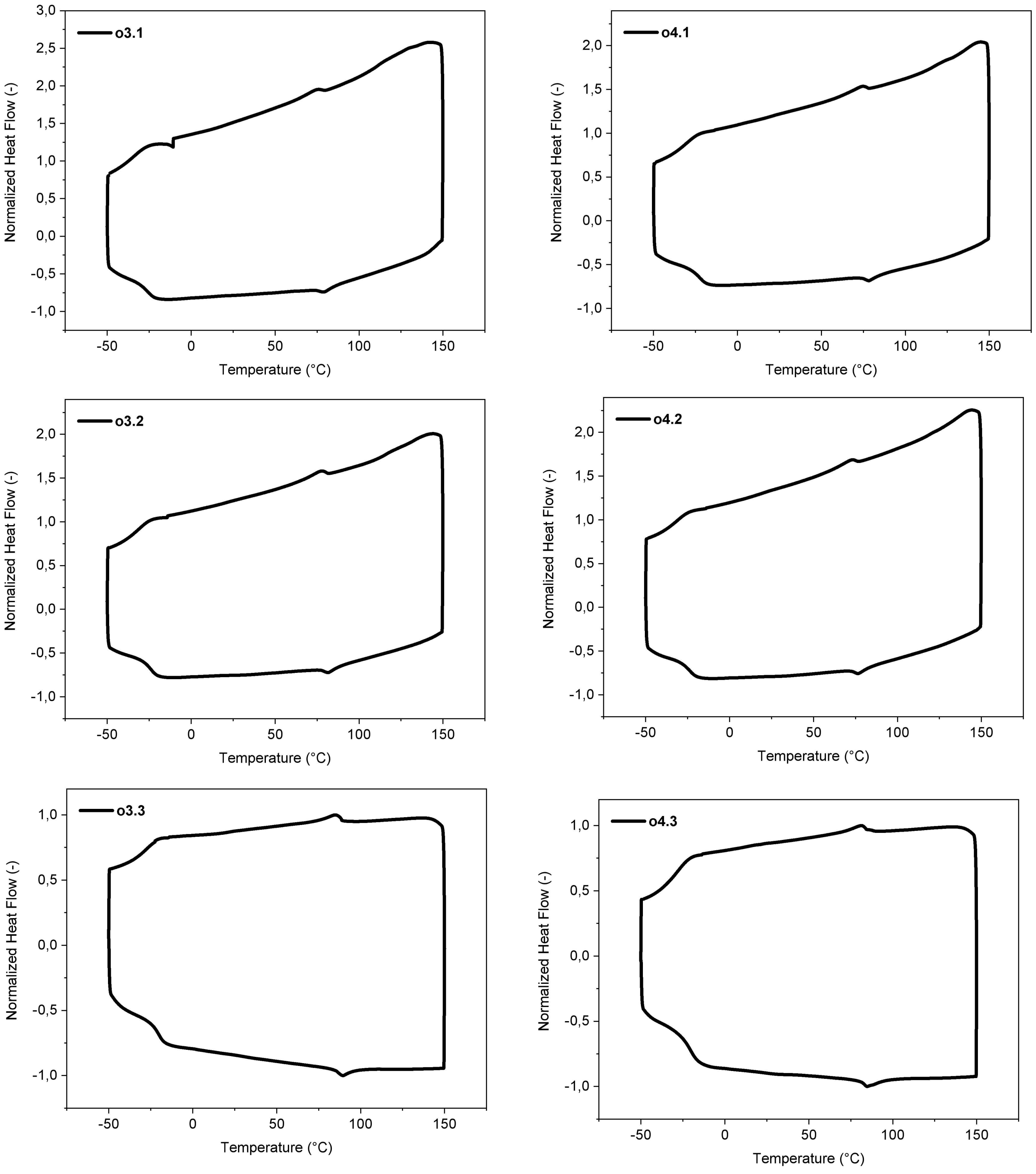

Figure S7. DSC-curves of the synthesized oligomers.

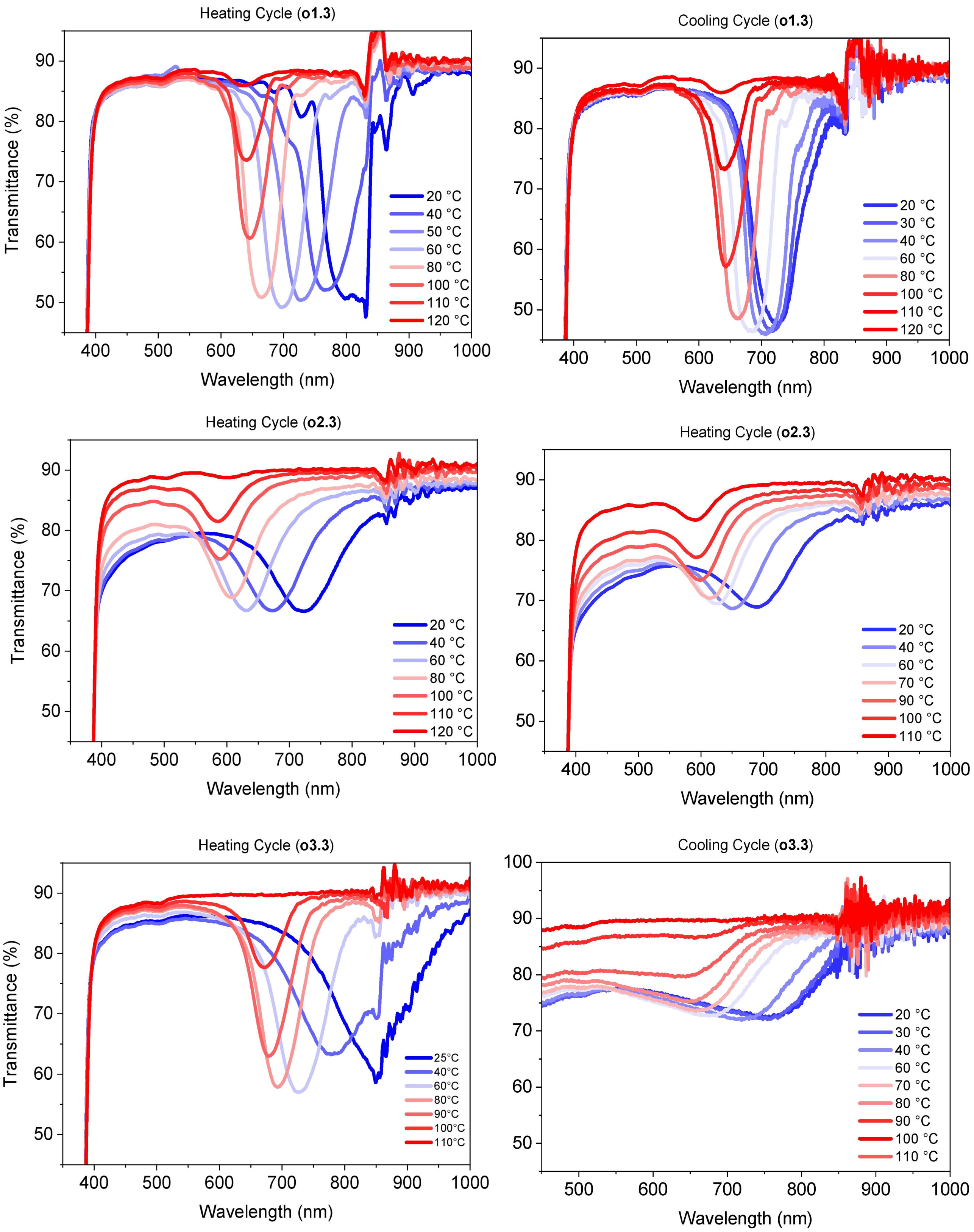

Figure S8. Temperature-dependent transmittance spectra of trimeric CLC oligomers. Both

heating and cooling cycles are shown.

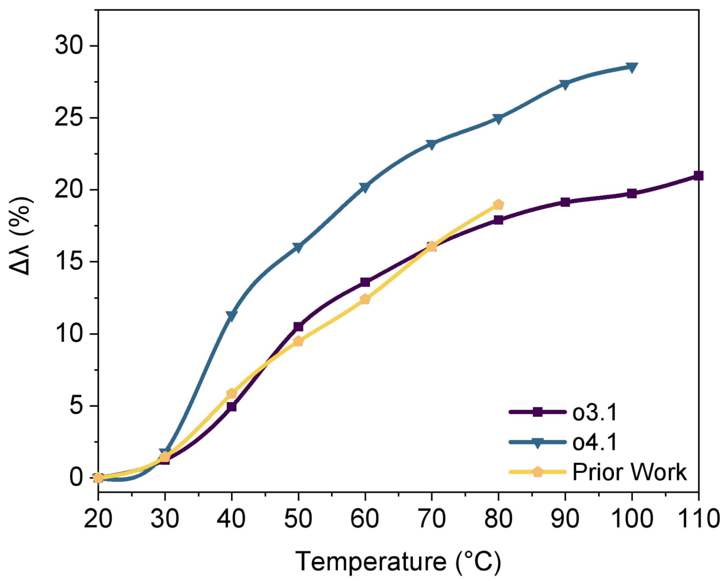

Figure S9. Normalized wavelength shifts with respect to temperature for **o3.1** and **o4.1** compared to previous work.[1] The previously reported oligomer has a similar composition as **o4.1**, with the same average length and diacrylate mesogen, but with monoacrylate mesogen shown in Figure S1 instead of **1** as endcapping molecule. It is apparent that the oligomers produced in this work have a response that equals or exceeds that of the previously reported oligomers.[1]

References

[1] Sentjens H, Lub J, Kragt AJJ, et al. Impact of Endcap Molecules on Temperature-Responsive Cholesteric Liquid Crystal Oligomers in Structural Color Stability and Hypsochromic Shift. Chem - A Eur J. 2024;202304236:1–12.

[2] Van Der Zande BMI, Lub J, Verhoef HJ, et al. Patterned retarders prepared by photoisomerization and photopolymerization of liquid crystalline films. Liq Cryst. 2006;33:723–737.

[3] Lub J, Broer DJ, Van Den Broek N. Synthesis and polymerization of liquid crystals containing vinyl and mercapto groups. Liebigs Ann. 1997;1997:2281–2288.

[4] Van Der Zande BMI, Roosendaal SJ, Doornkamp C, et al. Synthesis, properties, and photopolymerization of liquid-crystalline oxetanes: Application in transflective liquid-crystal displays. Adv Funct Mater. 2006;16:791–798.
